# Culture versus PCR for *Salmonella* Species Identification in Some Dairy Products and Dairy Handlers with Special Concern to Its Zoonotic Importance

**DOI:** 10.1155/2014/502370

**Published:** 2014-04-03

**Authors:** Mayada M. Gwida, Maha A. M. AL-Ashmawy

**Affiliations:** ^1^Department of Hygiene and Zoonoses, Faculty of Veterinary Medicine, Mansoura University, Mansoura 35516, Egypt; ^2^Department of Food Hygiene and Control, Faculty of Veterinary Medicine, Mansoura University, Mansoura 35516, Egypt

## Abstract

A total of 200 samples of milk and dairy products as well as 120 samples of dairy handlers were randomly collected from different dairy farms and supermarkets in Dakahlia Governorate, Egypt. The conventional cultural and serotyping methods for detection of *Salmonella* in dairy products were applied and the results were compared with those obtained by molecular screening assay using (*ttr* sequence). The obtained results revealed that 21% of milk and dairy products (42/200) were positive for *Salmonella* species using enrichment culture-based PCR method, while 12% of different dairy samples (24/200) were found to be positive for *Salmonella* species by using the conventional culture methods. Two stool specimens out of 40 apparently healthy dairy handlers were positive by the PCR method. Serotyping of *Salmonella* isolates revealed that 58.3% (14/24) from different dairy products were contaminated with *Salmonella* Typhimurium. We conclude that the enrichment culture-based PCR assay has high sensitivity and specificity for detection of *Salmonella* species in dairy products and handlers. High incidence of *Salmonella* Typhimurium in the examined dairy samples highlights the important role played by milk and dairy products as a vehicle in disease prevalence. Great effort should be applied for reducing foodborne risk for consumers.

## 1. Introduction

Foodborne diseases remain a major global public health concern. It does not only affect people's health and well-being, but it also has many economical drawbacks. In 2005, nearly 20% of acute diarrhea observed worldwide was attributed to bacterial enteropathogens. 1.8 million people die every year from diarrheal diseases mostly in developing countries. A great proportion of these cases can be due to contamination of food and drinking water [1]_._ *Salmonella*,* Shigella,* and* Campylobacter *are the principle pathogens responsible for 89% of bacterial gastroenteritis infections [2]. Salmonellosis is caused by the bacterium* Salmonella enterica*. Currently, there are more than 2500* Salmonella *serotypes [3]. The most common infectious* Salmonella* species are* Salmonella *Enteritidis and* Salmonella *Typhimurium which together account for three-quarters of all salmonellosis cases each year. The majorities of human salmonellosis cases have been associated with the consumption of raw or inadequately heat-treated dairy products [4]. The presence of* Salmonella *species in raw milk generally comes from feces of infected animals. Dairy cattle are the natural reservoirs of* Salmonella *species. Diagnosis of infected animals is difficult due to asymptomatic or subclinical infection and the fact that affected cows can shed as many organisms in their manure, providing an easy route of contamination during milking and milk processing [5].* Salmonella* presence in milk and some dairy products emerged as major public health concern to human consumers, causing great public health problem, as salmonellosis is an important zoonotic disease and being one of the most commonly reported causes of foodborne disease over the past century [6]. The most commonly used technique for* Salmonella* detection is the traditional microbiological technique. In spite of being the gold standard, these methods are generally labor- and time-consuming, requiring a minimum of 4–6 days, therefore, increasing the risk of uptake or transmitting pathogens [7]. Culture methods have also been reported to show poor sensitivity for low-level contamination with a high background of indigenous microflora in the samples, rendering the recovery of target organism difficult [8]. Polymerase chain reaction (PCR) and more recently real-time PCR assays have been developed for the detection of* Salmonellae* or specific serotypes in a variety of foods [9–11]. Several PCR assays have been developed by targeting various* Salmonella *genes, such as* 16S rRNA* [12],* agfA* [13],* viaB* [14], and virulence-associated plasmids [15]. In addition,* ttrBCA* sequences were specific for all* Salmonella* serovars [16]. In Egypt, salmonellosis remains a neglected zoonotic disease. In addition, there is lack of information available on the presence of* Salmonella* species in raw milk and some dairy products sold in Mansoura city at Dakahlia Governorate, Egypt. Therefore, the main objective of the present study was to screen the presence of* Salmonella* species in milk and some dairy products as well as some human samples to evaluate the role of milk and dairy products in transmitting such pathogenic bacteria to human beings and to assess the personal hygiene of dairy handlers as well as the potential hazards to consumers.

## 2. Methods

### 2.1. Sample Collection

A total of 200 samples of milk and dairy products (bulk farm milk, market raw milk, Damietta cheese, and kareish cheese, 50 each) were randomly purchased in their retail packs from different dairy farms and groceries located at Dakahlia Governorate, Egypt, in addition to 120 human samples consisting of hand swabs and stool specimens from dairy handlers as well as stool specimens from diarrheic patients; 40 each. The diarrheic patients attend the outpatient clinic of Gastrointestinal Tract Hospitals, Mansoura University, Dakahlia Governorate. The study was conducted in the period between December 2012 and May 2013. Swabs from human sources were transferred into sterile buffered peptone water (BPW). All samples were transported in a refrigerated box (4–8°C) to the laboratory where the bacteriological analyses were done immediately.

### 2.2. Sample Preparation

Twenty-five grams of collected cheese samples were weighed in a blender bag and homogenized with 225 mL of 0.1% BPW using a blender [17]. Additionally, the collected hand and stool swabs in BPW were subjected to the same laboratory diagnostic techniques in the same manner as done for dairy products [18, 19].

### 2.3. Microbiological Methods

Isolation and identification of* Salmonella *were performed using techniques recommended by US Food and Drug Administration [20]. Briefly, 25 gm or mL from the collected samples was preenriched with 225 mL of BPW and incubated for 24 hrs at 37°C. 0.1 mL of preenriched culture was transferred to 10 mL rappaport vassiliadis (RV) Broth (Oxoid) and incubated at 41°C for 18 to 24 hrs. Finally, loopful from the selective enrichment broth was inoculated onto xylose lysine deoxycholate (XLD) (Oxoid) agar and incubated at 37°C for (18 to 24) hrs. The incubation period could be prolonged up to 48 hrs for those not showing any growth during the 24 hrs of incubation. Characteristic* Salmonella* colonies from examined samples, having a slightly transparent zone of reddish colour and a black centre, were subcultured on nutrient agar slants, then incubated at 37°C for 24 hours, and then stored at 4°C until identification analyses were performed.

### 2.4. Identification and Characterization of Isolated* Salmonella* Strains

Each stored isolate was streaked onto XLD agar plate and incubated at 37°C for 24 hours. Suspected colonies were identified microscopically after Gram staining. Then, biochemical identification was performed using Kligler iron agar (KIA), Christensen's urea agar, Simmon citrate agar, lysine iron agar (LIA), Voges-Proskauer (VP), methyl red (MR), and indole tests [18].* Salmonella* strains confirmed by biochemical tests were differentiated serologically into serovars at the Central Laboratories of Egyptian Ministry of Health as previously described [21].

### 2.5. Molecular Assays

#### 2.5.1. Extraction of DNA by Thermal Cell Lysis of Suspended Bacteria from Enrichment Broth

DNA was extracted from the aliquot of enriched samples in BPW. A 15 mL of enrichment broth was transferred to centrifugal tube and was then centrifuged at 4000 rpm for 10 min in order to suspend cell pellets as much as we can obtain. The supernatant was discarded and the harvested cell pellet was resuspended in 1 mL sterile distilled water and transferred into 1.5 mL centrifuge tube and centrifuged at 14000 g for 10 min. The supernatant was discarded carefully. The pellet was resuspended in 100 *μ*L of sterile distilled water by vortexing. The tube was centrifuged again at 14000 g for 10 min, and the supernatant was discarded carefully. The pellet was resuspended once again in 100 *μ*L of sterile distilled water by vortexing and put in heat block at 95°C for 15 min. After heat treatment, the cell debris was pelleted by centrifugation at 14000 g for 10 min. The volume of the DNA containing supernatant was estimated by pipetting to a new microcentrifuge tube and varied from 40 to 60 mL, due to differences in removing the supernatant during the different washing steps of the DNA isolation method. The DNA was stored at –20°C until PCR assay was performed. An aliquot of 2 *μ*L of the supernatant was used as the template DNA in the PCR [22].

#### 2.5.2. Molecular Detection of* Salmonella* Species Using PCR

The DNA extracted from enrichment broth was screened by PCR using primers ttr6 and ttr4 for the amplification of a highly conserved DNA region (*ttr* sequence) specific for all* Salmonella* serovars according to Malorny et al. [16]. This region was encoded for tetrathionate reductase structural proteins. The ability to respire tetrathionate is likely to be significant within the life cycle of* Salmonella* spp. [23]. The sequences of the oligonucleotide primer sets used were (5′CTCACCAGGAGATTACAACATGG3′) as forward primer and (5′AGCTCAGACCAAAAGTGACCATC3′) as reverse primer and the expected size was 94 bp (Figure 2). PCR was performed with the Dream Taq Green PCR Master Mix (Fermentas). Cycling conditions were optimized at initial denaturation at 96°C for 1 min, followed by 35 cycles of denaturation at 96°C for 15 sec, annealing at 60°C for 60 sec, and elongation at 72°C for 15 sec with a final extension at 72°C for 1 min. PCR products were visualized using ethidium bromide stained 2% agarose gel electrophoresis. The separated PCR products were then visualized under UV light and photographed.

## 3. Results and Discussion

The present study was conducted to shed light on* Salmonella* species isolated from milk and dairy products.* Salmonella* species, as a marker of food products safety, is widely distributed foodborne pathogen [24]. The presence and growth of* Salmonella* in milk and some dairy products have been investigated because of their health significance.

**Figure 1:**
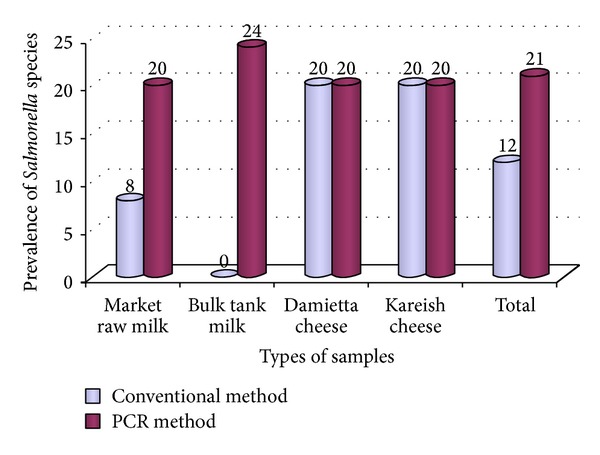
Prevalence of* Salmonella* species in the examined milk and dairy products samples.

**Figure 2:**
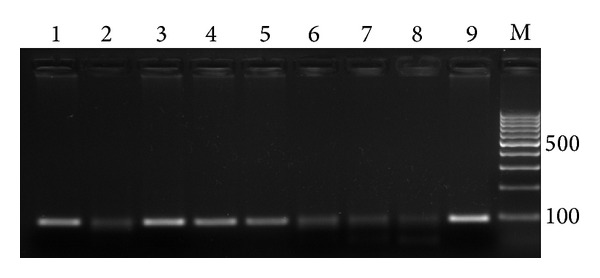
Agarose gel electrophoresis of ttr gene amplicon. lanes 1, 3, 4, and 5 are positive PCR product, lane 8 is negative control, lane 9 positive control, and lane 10 is DNA marker.

As shown in Figure 1, 21% (42/200) of dairy products were positive for* Salmonella* species using enrichment culture-based PCR method, while 12% (24/200) of different dairy samples were found to be positive for* Salmonella* species by using the conventional culture methods.

For market milk samples, 4 out of 50 with a percentage of 8% were positive for* Salmonella* species by conventional culture methods. Meanwhile, 10 out of 50 (20%) were positive by PCR assay.

Regarding the bulk tank milk, it was found that 12 samples (24%) were positive by PCR were culture negative.* Salmonella* species were not detected in any of the examined bulk tank milk samples by conventional culture methods. This result is in agreement with some researchers [25, 26] who failed to isolate* Salmonella* from their examined milk samples. On the contrary, high isolation rate (2.7%) was previously obtained by other researchers [27]. It appears, from our results, that PCR assay is superior to the conventional culture methods for the detection of* Salmonella* species. While PCR offers rapid, sensitive, and specific detection of pathogens, its major disadvantage lies in its inability to evaluate viability due to the presence of DNA in dead cells, whereas cultural methods would only detect and enumerate viable cells [28]. Likewise,* Salmonella* species was not isolated from farm milk samples, but 12 samples were PCR positive. It seems likely that the bulk tank milk contains many other organisms that may interfere with the growth of* Salmonella* in the enrichment medium, keeping the total number of* Salmonella* lower than the detection limit on plates. Additionally, the presence of other organisms on XLD selective agar plates may interfere with the production of H_2_S by* Salmonella* which is important for identification [29]. Fresh milk obtained from a healthy cow is virtually sterile, containing a low microbial load of less than 10^3^ CFU per milliliter. Pathogenic bacteria can gain access to the milk through three main sources: within the udder, outside the udder, and from the surface of equipment used for milk handling and storage [30].

Cheese is a well-known milk product which has gained great popularity throughout the world for its health promotion; it is ready-to-eat (RTE) food product that does not undergo any further treatment to ensure their safety before consumption. Soft cheese is considered the most popular Egyptian dairy product. It is regularly consumed in the daily life of Egyptian people. Their manufacture and handling techniques are still primitive and unhygienic [31]; therefore, contamination of cheese with foodborne pathogens may occur at several stages. Our findings showed that* Salmonella* species were isolated from 10 out of 50 examined samples, with a percentage of 20% and all samples positive by conventional culture method were also positive by using PCR method. The main sources of pathogenic bacteria in cheese were contaminated raw milk, food handlers, dust, utensils, and insects [32]. Raw milk contaminated with* Salmonella* spp. and introduced into dairy processing plant constitutes a risk to human health. Our findings were higher than those obtained in a previous report that isolated* Salmonella* spp. from Kareish cheese with a percentage of 3.33% [33]. However, several authors could not recover* Salmonella* species from Kareish cheese [34, 35]. Concerning the serotyping of 24* Salmonella* positive strains yielded by conventional culture methods (Table 1),* Salmonella enterica* subsp*. enterica* serovar Typhimurium was considered the major cause of* Salmonella* infection among the examined raw market milk and Damietta and Kareish cheese. 58.3% (14/24) of different dairy products were contaminated with* Salmonella* Typhimurium which poses great public health hazards. Consumption of contaminated milk and dairy products represents a common cause of human infection with salmonellosis.* Salmonellae*, particularly* Salmonella* Typhimurium and* Salmonella* Dublin, were commonly found in cattle and are excreted in the feces. That provides an easy route of contamination during milking and milk processing [36].

The results of market and bulk milk samples as well as both soft cheese samples did comply with neither Egyptian nor European standards, stating that milk and dairy products should be free from* Salmonella* [37–40]. National governments should provide more attention to ready-to-eat food as soft cheeses, from production time and throughout their expected shelf life. Many rapid, specific, and accurate protocols for detection should be provided and applied as well.

The overall percentage of* Salmonella* species in the total examined human samples was 1.7%. Two stool specimens out of 40 (5%) of the apparently healthy dairy handlers were positive by PCR assay. Asymptomatic infections with* Salmonella* species are thought to be common, although the proportion of patients who do not manifest disease is not known [41]. All collected hand swabs were found to be free from* Salmonella*. In contrast to our results, higher percentages of* Salmonella* among diarrheic people were previously reported by several authors [42–44] whose results were 13.6%, 80.5%, and 53.5%, respectively. From the obtained data, we cannot ignore the role played by the apparently healthy carriers in dissemination of infection; hence, it was of utmost importance to examine stool samples of apparently healthy persons (nondiarrheic stool samples) to clarify their role in shedding of bacterial pathogens.

## 4. Conclusion 

Rapid detection of pathogens in food is critical for the diagnosis of food poisoning and monitoring of food safety. Our findings represent the first report of using enrichment culture-based PCR method to detect* Salmonella* species in selected dairy products and handlers in Egypt and it can provide efficient and reliable results with high sensitivity and specificity. In our study, the dairy products sold in Dakahlia Governorate are contaminated with* Salmonella* species. These isolates constitute great public health hazards to consumers. National governments should apply periodical examination of dairy products to ensure consumers safety. Isolation of* Salmonella* species from apparently healthy dairy workers indicates bad hygienic standards and prerequisite regular worker inspection for surveillance of foodborne pathogens. Information on health hazards associated with contaminated dairy products should be provided to the public, so that consumption of untreated raw milk could be avoided.

## Figures and Tables

**Table 1:** Prevalence of *Salmonella *serovars among milk and dairy products.

Type of products	*Salmonella* species	Number	%
Market milk (*n* = 50)	*Salmonella *Typhimurium	**4**	**16.7**

Damietta cheese (*n* = 50)	Subsp. *III Arizona *serovar	**5**	**20.8**
	*Salmonella * Typhimurium	**5**	**20.8**

Kareish cheese (*n* = 50)	Subsp. *salamae* serovar	**3**	**12.5**
	Subsp. *III Arizona *serovar	**2**	**8.3**
	serovar Typhimurium	**5**	**20.8**

Total		**24**	**100**
